# Characteristics and outcomes of severe COVID-19 in hospitalized patients with cardiovascular diseases in the Amazonian region of Brazil: a retrospective cohort

**DOI:** 10.1038/s41598-022-23365-9

**Published:** 2022-11-02

**Authors:** Daniele Melo Sardinha, Carmem Aliandra Freire de Sá, Yan Corrêa Rodrigues, Joyce dos Santos Freitas, Ketan Brodeur, Rosane do Socorro Pompeu de Loiola, Karla Valéria Batista Lima, Ricardo José de Paula Souza e Guimarães, Luana Nepomuceno Gondim Costa Lima

**Affiliations:** 1 Programa de Pós-Graduação em Biologia Parasitária na Amazônia, Universidade do Estado do Pará and Instituto Evandro Chagas (PPGBPA/UEPA/IEC), Belém, Pará Brazil; 2 Programa de Pós-Graduação em Epidemiologia e Vigilância em Saúde, Instituto Evandro Chagas (PPGEVS/IEC), Ananindeua, Pará Brazil; 3 Programa de Pós-Graduação em Doenças Tropicais, Núcleo de Medicina Tropical, Universidade Federal do Pará (PPGDT/NMT/UFPA), Belém, Pará Brazil; 4 University of Michigan, Ann Arbor, MI USA; 5 Programa de Pós-Graduação em Biologia de Agentes Infeciosos e Parasitários, Universidade Federal do Pará (PPGBAIP/UFPA), Belém, Pará Brazil

**Keywords:** Cardiology, Risk factors, Signs and symptoms, Respiratory tract diseases

## Abstract

The northern region of Brazil is already vulnerable to other infectious diseases and it was no different in COVID-19. However, cardiovascular diseases still lead the causes of death. Thus, the objective of this study is to identify the clinical predictors and outcome of severe COVID-19 in hospitalized patients with and without CVD in this region of the Amazon. A retrospective cohort, referring to the notifications from January 1 to December 31, 2020, including cases confirmed by molecular testing. The study consisted of 9223 confirmed cases for COVID-19. Of these, 6011 (65.17%) did not have cardiovascular disease and 3212 (34.83%) had some cardiovascular disease. The significance of deaths was in the age group of < 1 to 59 CVD carriers (< 0.001). Predictor of mortality were invasive ventilation for patients with CVD, (OR 23,688 CI 18,180–30,866), followed by chronic kidney disease (OR 2442 CI 1568–3740), dyspnea (OR 2312 CI 1817–3941), respiratory distress (OR 1523 CI 1210–2919), cough (OR 1268 CI 1005–1599), Lower oxygen saturation 95% (OR 1281 CI 1039–1579), diabetes mellitus (OR 1267 CI 1050–1528) and age (OR 1051 CI 1044–1058). Carriers of CVD had a lower survival rate (< 0.0001). The order of the predictors of death differed among the non-carriers, as well as the high odds ratio in the predictors of CVD, only cough was an independent predictor. The age group under 59 years was associated with deaths. We also show the shorter survival in CVD carriers, as well as the higher cardiovascular morbidity rate than other studies in the literature.

## Introduction

COVID-19 is a respiratory disease that can develop into a severe systemic form, being directly and indirectly transmissible by droplets, it is caused by the novel SARS-CoV-2 coronavirus that emerged in China in December 2019 and reached the pandemic level in March 2021^1^. It still has a strong presence in Brazil, particularly in the northern region of the country, despite being the second year of the pandemic, its evolution was influenced by the lack of public policies and control measures aimed at combating the disease^2,3^.

Although the COVID-19 pandemic was responsible for 230,452 deaths in Brazil in 2020^4^, it did not surpass deaths from Cardiovascular Diseases (CVD), which amounted to 291,375 between March and December alone, the main cause being Acute Myocardial Infarction (AMI). Thus, the main cause of mortality and morbidity in Brazil and in the world is due to CVD^5,6^.

In this context, the northern region of Brazil has different characteristics from other regions, located in the Amazon Forest, with a diversity of indigenous peoples, environmental mercury contamination, fires, mining regions, large land area and rural populations with lack of access to high complexity health care services. Due to these characteristics, infectious diseases are usually more lethal in this region of the country. They are also associated with higher morbidity rates in the population because of physiological changes, neurological and autoimmune diseases due to exposure to mercury, and a lack of diagnoses and epidemiological surveillance^7–11^.

With regard to CVD, the literature already describes that it is an important risk factor for severity and lethality in Severe Acute Respiratory Syndromes (SARS) of viral etiologies such as influenza, SARS-CoV, MERS-CoV and the new coronavirus SARS-CoV-2^12–16^. A study in northern Italy compared CVD carriers and non-carriers on clinical features and outcome in SARS from SARS-CoV-2 and found that hospitalized patients with concomitant heart disease and COVID-19 have an extremely poor prognosis compared to individuals without a history heart disease, with higher mortality, thromboembolic events and septic shock rates^17^. However, another study in Iran, when assessing the same aspects and adjusting for age, CVD had no significance in mortality from COVID-19, only Diabetes^18^.

A study in 2020 in six major Brazilian capitals with the highest fatalities from COVID-19 showed that deaths from CVD increased when compared to 2019, highlighting the capitals in the north of the country, (132%) Manaus and (126%) Belém, were the leading cause of death in all of the evaluated capitals^19^.

No studies in Brazil or in the Amazon have been carried out to assess the clinical outcome of severe COVID-19 and factors associated with people with cardiovascular disease. We saw that the characteristics of this population are not similar to other regions of Brazil or the world. Thus, the objective of this study is to identify the clinical predictors and outcome of severe COVID-19 in hospitalized patients with and without CVD in this region of the Amazon based on data from the epidemiological surveillance of acute and severe respiratory syndromes in Brazil.

## Materials and methods

### Type of study and ethical issues

A longitudinal retrospective cohort study based on data from the epidemiological surveillance of acute and severe respiratory syndromes in Brazil publicly available on the website (https://opendatasus.saude.gov.br/).

According to Resolution No. 510, OF APRIL 7, 2016, highlights article II, which establishes that searches that use publicly accessible information, according to Law no. 12527 of November 18, 2011; III—searches that use information from the public domain and V—searches in databases, whose information is aggregated, without the possibility of individual identification. They must not be registered or evaluated by the Ethics and Research Committee (CEP/CONEP) system^20,21^. Thus, these types of studies are not recommended to be submitted for ethical evaluation and can be carried out freely, since publicly available data do not contain information such as the participant's name, telephone number and address.

This study followed the guidelines of the Report of Observational Studies in Epidemiology (STROBE)^22^.

All information used in this study was obtained from sources with universal public access and, therefore it was not submitted to a research ethics committee for approval. All methods we used to analyze our data were carried out in accordance with relevant guidelines and regulations.

### Place of study

**Figure 1:**
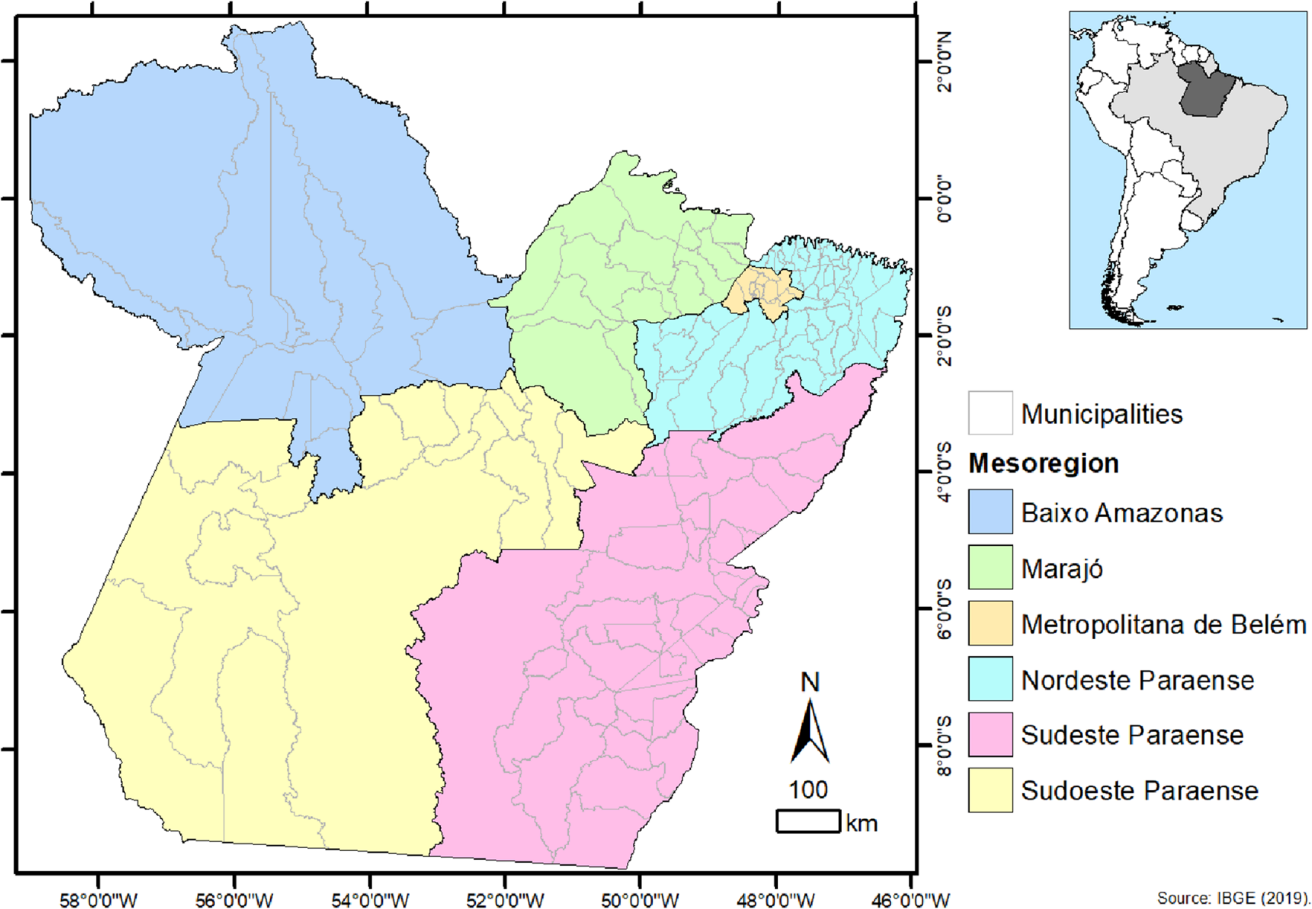
Spatial location of Pará, mesoregions and municipalities, Brazil. Source: Sardinha et al.^25^. Software: ArcGis 10.4 (https://www.arcgis.com/).

The survey was conducted referring to notified cases and residents in the state of Pará in northern Brazil in the eastern territory of the Amazon (Fig. 1). Pará is the second largest state in terms of land area in the country with an area of 1,245,870,798 km^2^ and has an estimated population of 8,690,745 inhabitants in 2020 with a Human Development Index (HDI) of 0.646. The state has six Mesoregions comprising 22 Microregions, in a total of 144 municipalities, and its capital is Belém^23^. The territory of Pará comprises the largest tropical forest in the world, the Amazon. The relief is low and flat; 58% of the territory is below 200 m. The altitudes above 500 m are in the following mountains: Serra dos Carajás, Serra do Cachimbo and Serra do Acari^24^.

The cases were selected in reference to their notification dates from March 1, 2020 to December 31, 2020, with the epidemiological investigations completed and the outcome of each case completed.

### Participant selection

The database refers to the flu epidemiological surveillance platform (SIVEP-GRIPE) which carries out surveillance of acute and severe respiratory syndromes, and is available on the OpenDataSUS platform of the Brazilian Ministry of Health.

The cohort zero-time was defined by the date of hospitalization, and the delta-time (∆T) corresponded to the period from the date of hospitalization to the outcome (cure or death) for cases with diagnostic confirmation of SARS by COVID-19 by the Real-Time Polymerase Chain Reaction (RT-PCR). The follow-up time was until the outcome.

SARS is defined for individuals with Flu Syndrome (FS) who present with: dyspnea/respiratory discomfort OR persistent pressure in the chest OR O2 saturation of less than 95% in room temperature OR a bluish discoloration of the lips or face. (FS: Individual with acute respiratory condition, characterized by at least two (2) of the following signs and symptoms: fever (even if referred), chills, sore throat, headache, cough, runny nose, olfactory or taste disturbances). For reporting purposes in the SIVEP-GRIPE, hospitalized SARS cases or deaths due to SARS regardless of hospitalization should be considered^26^.

The definition of cardiovascular diseases was according to the COVID-19 Brazilian epidemiological surveillance guide, which cites the following diseases: Myocardiopathies of different etiologies (heart failure, ischemic myocardiopathy, etc.) Hypertension; Cerebrovascular disease^27^.

**Figure 2:**
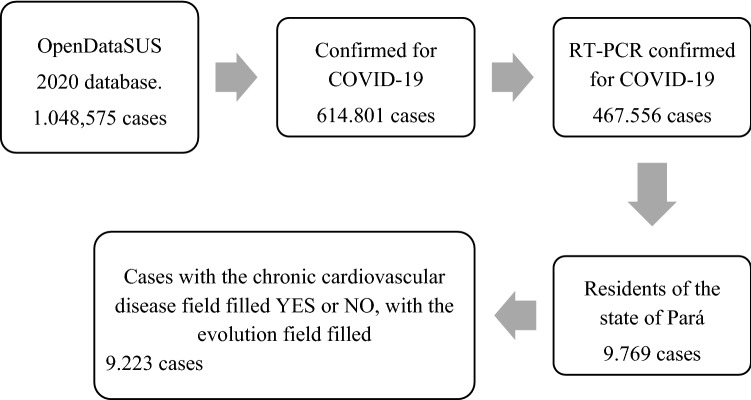
Selection flowchart of participants.

Data was downloaded for the 2020 year, notified from January 1st to December 31st, 2020. The selection details until the final population is described in the flowchart below (Fig. 2).

For the eligibility criteria, confirmed cases for COVID-19 by the RT-PCR criterion and residents of the state of Pará with field 36 of the investigation form filled in 1 (yes) or 2 (no) with chronic cardiovascular disease, those with the outcome were selected, field 74 filled in. Cases that did not meet the eligibility criteria were excluded^26^.

### Data collection

The data were made available in Excel format with the variables referring to the notification form of acute and severe respiratory syndromes 25. The data includes demographic, epidemiological and investigation outcome variables, however the database made available for this study does not have the following variables: Register of individual, name, telephone, street, house number, neighborhood, Postal Address Code (zip code) and telephone.

Data were downloaded on August 2, 2021 and the selection of participants was carried out, after which the variables were extracted: sex (item 8), age (item 10), municipality of residence (item 19), signs and symptoms (item 35), has risk factors/comorbidities (item 36), received flu vaccine (item 37), date of admission (item 43) was admitted to the ICU (item 47), used ventilatory support (item 50) RT-PCR result /other method by Molecular Biology (item 64) final classification (item 72) evolution (item 74) and date of discharge or death (item 75).

The study is subject to information bias, due to the use of secondary data from epidemiological surveillance, with the possibility of diagnostic and recording errors, and/or the inability to control for possible confounding variables. However, it is assumed that the present information bias is of the non-differential type. The form has the variable with chronic cardiovascular disease to mark YES or NO, thus it does not specify which cardiovascular disease the individual has.

We chose not to include the 2021 cases as the data are still being processed in the system or are still in the process of epidemiological investigation. In Brazil, epidemiological surveillance data are usually reliable after the data processing period, which basically represents 12 months after notification.

### Data analysis

The primary outcome of the study was to evaluate the characteristics of COVID-19 in hospitalized patients with CVD 3212 (34.83%), clinical, comorbidity and outcome characteristics were compared with 6011 (65.17%) non-carriers.

A cardiovascular morbidity rate in the general population (number of CVD carriers/all hospitalized cases × 100) was performed to estimate the incidence of cardiovascular comorbidities in this study. Likewise, the overall lethality rate (number of deaths/all cases × 100) was performed only in patients with CVD (deaths with CVD/cases with CVD × 100) and without CVD (deaths without CVD/cases without CVD × 100), as well as by age group to measure lethality in each group and observe differences. The chi-square test of adherence was applied to identify whether the lethalities of the groups have statistical significance in relation to the difference between them. The results were presented in tables.

Statistical analysis was performed using the Statistical Package for Social Sciences 20 program (SPSS—https://www.ibm.com/analytics/spss-statistics-software). Bivariate analysis from the Chi-square statistical tests for the independence test (2X2 table) and Fisher exact test (L × C Contingency Table 2 × 2) in values less than < 5. The Odds Ratio for the significant variables (< 0.05) was also performed, associating the significant variables between patients with CVD and non-carriers, analyzing the general and deaths. The results were presented in tables.

Binary logistic regression with the dependent variable death was performed for non-CVD patients and another model for CVD patients. In the univariate regression model with the independent covariates, age, sex, signs and symptoms, comorbidities and outcome. The multivariate regression model was performed with the significant covariates in the univariate model < 0.05, the multivariate model was adjusted by the 2 log likelihood—R2 Nagelkerke—Hosmer and lemeshow tests.

We performed the ROC curve (Receiver Operating Characteristic Curve) in the two adjusted multivariate models. The ROC curve measures the predictive capacity of the proposed model, through the predictions of sensitivity and specificity in association with the logistic model. This technique serves to visualize, organize and classify the model based on predictive performance. Regarding the interpretation of the ROC curve, it is considered that the larger the area under the curve, the greater the accuracy of the method, in this case, score and calculation of the probability of death. A suitable model is the one closest to 100% of the graph area; curves that occupy 50% or less of the graph area indicate that the accuracy of the model is not greater than the result that would be obtained by chance^28^.

Survival analysis was performed using the outcome survival (success) and death (failure), considering the date of onset of hospitalization, date of death and date of hospital discharge, comparing with the dependent variable being a CVD carrier, by the Kaplan method-Meier.

For all tests, the alpha level of significance was set at 0.05.

### Methodological guidelines

This study followed the guidelines of the Streng guideline recommendations thening the Reporting of Ob-servational Studies in Epidemiology (STROBE). As well as following the editorial and publication policies of the Nature Research journals https://www.nature.com/srep/journal-policies/editorial-policies.

## Results

The study consisted of 9223 confirmed cases for COVID-19 by the molecular method of RT-PCR and with criteria for defining SARS who were hospitalized in 2020. Of these, 6011 (65.17%) did not have cardiovascular disease and 3212 (34.83%) had some cardiovascular disease. Thus, the cardiovascular morbidity rate in this study was (34.83%).

The lethality in this study was 4213 (45.68%) higher than the overall cardiovascular morbidity rate, whereas lethality only in patients with CVD represented 1783 (55.51%) higher than the general lethality, and the lethality of non-carriers was 2430 (40.42%) lower than general and for CVD patients (Table 1).

**Table 1:** Lethality rate by age group, hospitalized by COVID-19, general, with and without cardiovascular disease in a region of the eastern Amazon in the first year of the pandemic, Pará state, Brazil, 2021.

General				
	Survivor	Death	Total	Lethality rate
**Age group**				
< 1	67	18	85	21.18
1–5	74	6	80	7.50
6–19	100	24	124	19.35
20–59	3012	1057	4069	25.98
> 60	1757	3108	4865	63.88
Total	5010	4213	9223	45.68
**With CVD**				
< 1	6	4	10	40.00
1–5	2	1	3	33.33
6–19	0	2	2	100.00
20–59	568	297	865	34.34
> 60	853	1479	2332	63.42
Total	1429	1783	3212	55.51
**No CVD**				
< 1	61	14	75	18.67
1–5	72	5	77	6.49
6–19	100	22	122	18.03
20–59	2444	760	3204	23.72
> 60	904	1629	2533	64.31
Total	3581	2430	6011	40.43

Difference in lethality by age group				
	General	With CVD	No CVD	*p-value
< 1	21.18	40	18.67	< 0.001
1–5	7.5	33.33	6.49	< 0.001
6–19	19.35	100	18.03	< 0.001
20–59	25.98	34.34	23.72	< 0.001
> 60	63.88	63.42	64.31	0.997
General	45.68	55.51	40.42	0.288

Source: OpenDataSUS, 2021.

*Chi-square test of adherence.

The mean age of all cases was 58 years and a median of 61, with a minimum of 0 and a maximum of 114 years and standard deviation of 20. In relation to cases without cardiovascular disease, the mean age was 54 years, median of 55, minimum of 0 and maximum of 114, with a standard deviation of 21. Among patients with cardiovascular diseases, the average age was 67 years, median 69, minimum of 0 and maximum of 101, with a standard deviation of 15.

In the lethality rate by age group, it was shown that in those over 60 years the rates were similar in the groups, in general, with and without CVD, influenced by the fact that advanced age is already an independent risk factor, as well as the presence of other comorbidities in this age group that also influence death. However, in the other age groups, all had statistical significance of greater difference in the lethality rate in CVD patients (< 1 to 59 years—p-value < 0.001) showing that CVD is an independent risk factor for death in COVID-19 severe among hospitalized patients (Table 1).

Regarding gender, most were male, overall 5472 (59.3%), 3684 (59.6%) without cardiovascular disease and 1888 (58.2) with cardiovascular disease, in the V.S. with and without cardiovascular comorbidity there was no difference (p-0.436). Regarding symptoms associated with patients with cardiovascular diseases, dyspnea 2573 (p- < 0.001–80.1%), respiratory distress 2379 (p-0.001–74.1%), O2 saturation < 95% 2081 (p- < 0.001–64.8%) and other symptoms 871 (p-0.003–27.1%). Fever 4794 (p- < 0.001–79.8%) and sore throat 2192 (p- < 0.001–36.5%) were associated with those without cardiovascular disease (Table 2).

**Table 2:** Clinical characteristics, comorbidities and outcome of cases hospitalized for COVID-19, total, without cardiovascular disease and with cardiovascular disease in a region of the eastern Amazon in the first year of the pandemic, state of Pará, Brazil, 2021.

Variables	Total (9223)	%	Cardiovascular disease				^#^P-Value	^&^Odds ratio	CI*
			No (6011)	%	Yes (3212)	%			
Male	5472	59.3	3584	59.6	1888	58.2	0.436		
Female	3751	40.7	2427	40.4	1324	41.2	0.436		
Fever	7228	78.4	4794	79.8	2434	75.8	< 0.001	0.794	0.717–0.880
Cough	7341	79.6	4816	80.1	2525	78.6	0.088		
Sore throat	3121	33.8	2192	36.5	929	28.9	< 0.001	0.709	0.646–0.778
Dyspnea	7187	77.9	4614	76.8	2573	80.1	< 0.001	1.209	1.097–1.355
Respiratory discomfort	6628	71.9	4249	70.7	2379	74.1	0.001	1.184	1.075–1.304
O2 saturation < 95%	5384	58.4	3303	54.9	2081	64.8	< 0.001	1.509	1.381–1.648
Diarrhea	1462	15.9	930	15.5	532	16.6	0.178		
Vomiting	743	8.1	506	8.4	237	7.4	0.084		
Other symptoms	2332	25.3	1461	24.3	871	27.1	0.003	1.159	1.051–1.278
Chronic hematologic disease	59	0.6	35	0.6	24	0.7	0.341		
Puerperal	17	0.2	13	0.2	4	0.1	0.447**		
Diabetes	1973	21.4	787	13.1	1186	36.9	< 0.001	3.886	3.503–4.310
Chronic liver disease	55	0.6	26	0.4	29	0.9	0.007	2.097	1.233–3.567
Asthma	175	1,9	126	2.1	49	1.5	0.065		
Down syndrome	21	0.2	13	0.2	8	0.2	0.820		
Chronic neurological disease	203	2.2	116	1.9	87	2.7	0.017	1.415	1.608–1.874
Other chronic lung disease	216	2.3	119	2	97	3	0.002**	1.542	1.175–2.023
Immunodeficiency/immunodepression	260	2.8	194	3.2	66	2.1	0.001	0.629	0.473–0.835
Chronic kidney disease	278	3	121	2	157	4.9	< 0.001**	2.502	1.966–3.184
Obesity	165	1.8	78	1.3	87	2.7	< 0.001	2.118	1.555–2.883
Another comorbidity	1414	15.3	393	6.5	1021	31.8	< 0.001	6.662	5.871–7.559
Influenza vaccine in the last year	1042	11.3	585	9.7	457	14.2	< 0.001	1.539	1.350–1.753
Admitted to the ICU	3244	35.2	1881	31.3	1363	42.4	< 0.001	1.619	1.481–1.769
Invasive ventilation	2597	28.2	1480	24.6	1117	34.8	< 0.001	1.632	1.487–1.792
Non-invasive ventilation	3074	33.3	1955	32.5	1119	34.8	0.026	1.109	1.013–1.214
Did not use ventilation	3552	38.5	2576	42.9	976	30.4	< 0.001	0.582	0.531–0.637
Death	4213	45.7	2430	40.4	1783	55.5	< 0.001	1.839	1.686–2.005

Source: OpenDataSUS, 2021.

^#^Chi-square.

**Fisher test.

^&^Odds ratio from bivariate analysis.

*Confidence Interval (95%).

Regarding comorbidities and risk factors, diabetes was significant in patients with CVD (p-< 0.001–36.9%), as well as other comorbidities, chronic liver disease (p-0.007–0.9%), chronic neurological disease (p- − 0.017 to 2.7%), other chronic lung disease (p-0.002–3%), chronic kidney disease (p-< 0.001–4.9%), obesity (p < 0.001–2.7%), other comorbidity (p < 0.001–31.8%). Immunodeficiency/Immunodepression (p-0.01–2.1%) was associated with non-CVD carriers (Table 2).

Regarding outcomes, those vaccinated against influenza in the last year were significant in patients with CVD (p < 0.001–14.2%), and admitted to the Intensive Care Unit (ICU) (p < 0.001–42.4%), invasive ventilation (p < 0.001–34.8%), non-invasive ventilation (p-0.026–34.8%) and death (p < 0.001–55.5%). Cases that did not use ventilation (p < 0.001–30.4%) were associated with those without CVD (Table 2).

In Table 3, the comparison of variables was performed only in deaths, with and without CVD, showing the association of the following signs and symptoms with CVD patients, Cough (p-0.002–78.2%), Dyspnea (p- 0.023–88.4%), Respiratory discomfort (p-0.044–81.2%), O2 Saturation < 95 (p-0.002–74.5%), Diarrhea (p-0.001–13.2%) and others (p < 0.001–21.8%). About comorbidities and associated risk factors, Diabetes (p < 0.001–39.3%), Chronic Kidney Disease (p < 0.001–6.4%), Obesity (p-0.004–2.6%), other (p < 0.001–29.5%) Hospitalized in the ICU (p-0.001–63.9%), Invasive ventilation (p-0.016–58.3%). Those associated with non-CVD carriers were patients who did not use ventilation (p < 0.001–14.8%) and Immunodeficiency/Immune depression (p < 0.001–2.2%).

**Table 3:** Clinical characteristics, comorbidities and outcome of deaths from COVID-19 without cardiovascular disease and with cardiovascular disease in a region of the eastern Amazon in the first year of the pandemic, Pará state, Brazil, 2021.

Variables	Deaths				^#^p-value	^&^Odds ratio	CI**
	Without CVD* (2430)	%	With CVD* (1783)	%			
Male	1510	62.1	1103	61.9	0.872		
Female	920	37.9	680	38.1	0.872		
Fever	1828	75.2	1316	73.8	0.299		
Cough	1800	74.1	1394	78.2	0.002	1.254	1.086–1.449
Sore throat	664	27.3	456	25.6	0.217		
Dyspnea	2091	86	1577	88.4	0.023	1.241	1.031–1.493
Respiratory discomfort	1912	78.7	1448	81.2	0.044	1.171	1.004–1.365
O2 saturation < 95%	1703	70.1	1329	74.5	0.002	1.250	1.089–1.434
Diarrhea	240	9.9	236	13.2	0.001	1.392	1.150–1.685
Vomiting	142	5.8	107	6	0.843		
Other symptoms	383	15.8	388	21.8	< 0.001	1.487	1.271–1.739
Chronic hematologic disease	23	0.9	15	0.8	0.745		
Puerperal	5	0.2	3	0.2	1.000**		
Diabetes	430	17.7	701	39.3	< 0.001	3.013	2.617–3.470
Chronic liver disease	16	0.7	17	1	0.294		
Asthma	30	1.2	20	1.1	0.775		
Down syndrome	6	0.2	4	0.2	1.000**		
Chronic neurological disease	70	2.9	54	3	0.783		
Other chronic lung disease	81	3.3	72	4	0.243		
Immunodeficiency/immunodepression	112	4.6	40	2.2	< 0.001	0.475	0.329–0.685
Chronic kidney disease	72	3	114	6.4	< 0.001	2.237	1.654–3.025
Obesity	33	1.4	47	2.6	0.004	1.967	1.255–3.082
Another comorbidity	183	7.5	526	29.5	< 0.001	5.138	4.284–6.163
Influenza vaccine in the last year	175	7.2	221	12.4	< 0.001	1.823	1.480–2.246
Admitted to the ICU	1431	58.9	1140	63.9	0.001	1.238	1.091–1.404
Invasive ventilation	1324	54.5	1039	58.3	0.016	1.167	1.031–1.320
Non-invasive ventilation	621	25.6	480	26.9	0.321		
Did not use ventilation	485	20	264	14.8	< 0.001	0.697	0.591–0.821

Source: OpenDataSUS, 2021.

^#^Chi-square.

**Fisher test.

^&^Odds ratio from bivariate analysis.

*Confidence Interval (95%).

Regarding lethality predictors, in the adjusted multivariate logistic regression model in cases without CVD (Table 4), we showed that invasive ventilation was the main predictor of death (OR 22,278 CI 18,229–27,225) increasing chances of death by twenty-two times, followed by chronic hematologic disease (OR 5170 CI 2120–12,609), immunodeficiency (OR 2049 CI 1415–3966), chronic kidney disease (OR 1988 CI 1239–3189), Other chronic lung disease (OR 1867 CI 1129–3087) dyspnea (OR 1715 CI 1429–2058), O_2_ saturation < 95% (OR 1351 CI 1154–1582), diabetes mellitus (OR 1336 CI 1100–1623), respiratory distress (OR 1327 CI 1115–1578) and age (OR 1051 CI 1047–1056).

**Table 4:** Odds ratio for death in COVID-19 cases hospitalized without CVD in an eastern Amazon region in the first year of the pandemic, Pará state, Brazil, 2021.

	Univariate regression		CI** for OR		Multivariate regression		CI** for OR	
	p-value	OR*	Lower	Upper	p-value	OR*	Lower	Upper
Invasive ventilation	< 0.001	22.713	16.672	30.943	< 0.001	22.278	18.229	27.225
Chronic hematologic disease	< 0.001	4.98	2.03	12.217	< 0.001	5.170	2.120	12.609
Chronic liver disease	0,122	2.113	0.82	5.449				
Immunodeficiency	< 0.001	2.042	1.411	2.955	< 0.001	2.049	1.415	2.966
Chronic kidney disease	0.007	1.934	1.202	3.113	0.004	1.988	1.239	3.189
Other chronic lung disease	0.011	1.919	1.158	3.178	0.015	1.867	1.129	3.087
Dyspnea	< 0.001	1.731	1.441	2.079	< 0.001	1.715	1.429	2.058
Chronic neurological disease	0,117	1.492	0.905	2.458				
O2 saturation < 95%	< 0.001	1.357	1.158	1.592	< 0.001	1.351	1.154	1.582
Diabetes mellitus	0.003	1.35	1.111	1.64	0.004	1.336	1.100	1.623
Respiratory discomfort	0.002	1.325	1.113	1.576	0.001	1.327	1.115	1.578
Obesity	0.684	1.141	0.606	2.147				
Down syndrome	0.892	1.091	0.311	3.832				
Age	< 0.001	1.051	1.047	1.055	< 0.001	1.051	1.047	1.056
Fever	0.919	1.01	0.837	1.218				
Vomiting	0.787	0.963	0.73	1.27				
Male gender	0.352	0.934	0.808	1.079				
Sore throat	0.003	0.793	0.679	0.926	0.002	0.789	0.677	0.919
Asthma	0.309	0.756	0.442	1.295				
Cough	< 0.001	0.705	0.584	0.851	< 0.001	0.708	0.591	0.850
Diarrhea	< 0.001	0.664	0.535	0.824	< 0.001	0.659	0.536	0.811
Admitted to the ICU	0.822	0.97	0.744	1.264				
Constant	< 0.001	0.013			< 0.001	0,012		

Source: Ministry of Health, Sivep-Gripe/OpenDataSUS, 2020.

Multivariate (2 log likelihood 4959,361^a^—p-value < 0.01)—(R^2^ Nagelkerke = 0.551)—(Hosmer and lemeshouw test—p-value 0.193).

*Confidence Interval (95%).

**Odds Ratio.

However, in the adjusted multivariate logistic regression analysis, the main predictor of mortality were invasive ventilation for patients with CVD (Table 5), being the main one. Similar to the analysis of non-carriers, but with a higher odds ratio (OR 23,688 CI 18,180–30,866), with a risk of death around twenty-four and a half times higher, followed by chronic kidney disease (OR 2442 CI 1568–3740), dyspnea (OR 2312 CI 1817–3941), respiratory distress (OR 1523 CI 1210–2919), cough (OR 1268 CI 1005–1599), O_2_ Saturation < 95% (OR 1281 CI 1039–1579), diabetes mellitus (OR 1267 CI 1050–1528) and age (OR 1051 CI 1044–1058). When we compared the predictors of the models, in cases without CVD and with CVD, we showed that in cases without CVD there were more predictors such as chronic hematologic disease, immunodeficiency, which were absent predictors in patients with CVD. Regarding the difference in the risk ratio in the predictors that were similar in the two multivariate models, in patients with CVD the odds ratios were higher, showing that CVD potentiates independent mortality predictors in COVID-19 in hospitalized patients, as well as cough, which has been shown to be associated with death in CVD patients.

**Table 5:** Odds ratio for death in COVID-19 cases hospitalized with CVD in the eastern Amazon region in the first year of the pandemic, Pará state, Brazil, 2021.

	Univariate regression		CI** for OR		Multivariate regression		CI** for OR	
	p-value	OR*	Lower	Upper	p-value	OR*	Lower	Upper
Invasive ventilation	< 0.001	19.841	13.728	28.675	< 0.001	23.688	18.180	30.866
Chronic kidney disease	< 0.001	2.342	1.509	3.636	< 0.001	2.422	1.568	3.740
Dyspnea	< 0.001	2.317	1.820	2.949	< 0.001	2.312	1.817	2.941
Down syndrome	0.470	1.890	0.337	10.599				
Respiratory discomfort	< 0.001	1.517	1.204	1.912	< 0.001	1.523	1.210	1.919
Immunodeficiency	0.257	1.441	0.767	2.708				
Chronic hematologic disease	0.487	1.435	0.518	3.975				
Cough	0.037	1.292	1.016	1.643	0.046	1.268	1.005	1.599
O2 saturation < 95%	0.024	1.274	1.032	1.573	0.020	1.281	1.039	1.579
Diabetes mellitus	0.012	1.273	1.054	1.538	0.014	1.267	1.050	1.528
Admitted to the ICU	0.212	1.209	0.897	1.629				
Other chronic lung disease	0.586	1.172	0.663	2.071				
Obesity	0.850	1.056	0.601	1.856				
Age	< 0.001	1.051	1.044	1.058	< 0.001	1.051	1.044	1.058
Fever	0.933	1.010	0.803	1.270				
Chronic neurological disease	0.840	0.944	0.537	1.658				
Vomiting	0.601	0.910	0.641	1.294				
Chronic liver disease	0.802	0.888	0.352	2.244				
Sore throat	0.054	0.816	0.664	1.003	0.020	0.789	0.645	0.964
Male gender	0.018	0.798	0.662	0.962	0.006	0.772	0.642	0.927
Diarrhea	0.064	0.790	0.615	1.014				
Asthma	0.073	0.491	0.225	1.069				
Constant	< 0.001	0.005			< 0.001	0.006		

Source: Ministry of Health, Sivep-Gripe/OpenDataSUS, 2020.

Multivariate (2 log likelihood 2892,716^a^—p-value < 0.01)—(R^2^ Nagelkerke = 0.505)—(Hosmer and lemeshouw test—p-value 0.446).

*Confidence Interval (95%).

**Odds Ratio.

**Figure 3:**
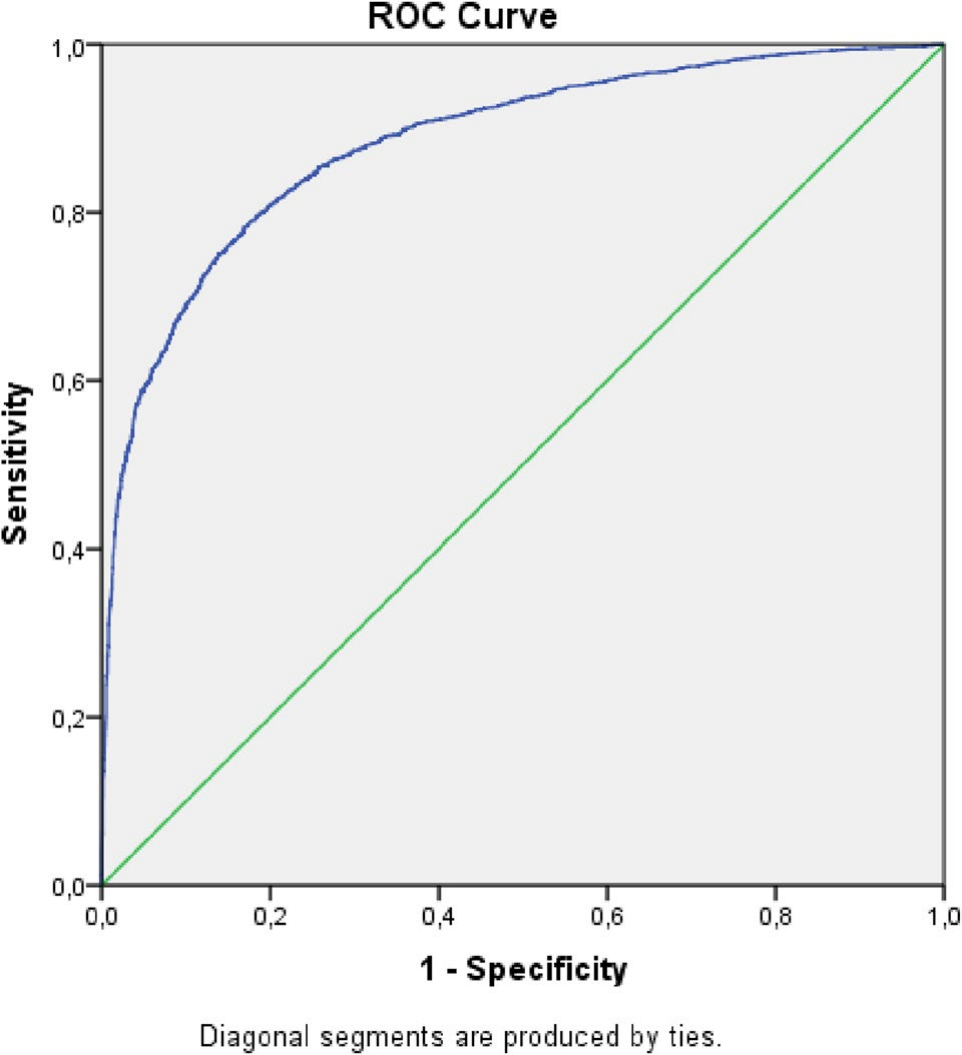
ROC curve of sensitivity and specificity of the multivariate model without CVD. Area Under the Curve, Test Result Variable(s): Predicted probability (area 0.884) (Std. error 0.04) (p-value < 0.01) (CI—0.875–0.892). Sensitivity (97.9%), Specificity (74.1%). Source: Ministry of Health, Sivep-Gripe/OpenDataSUS, 2020.

**Figure 4:**
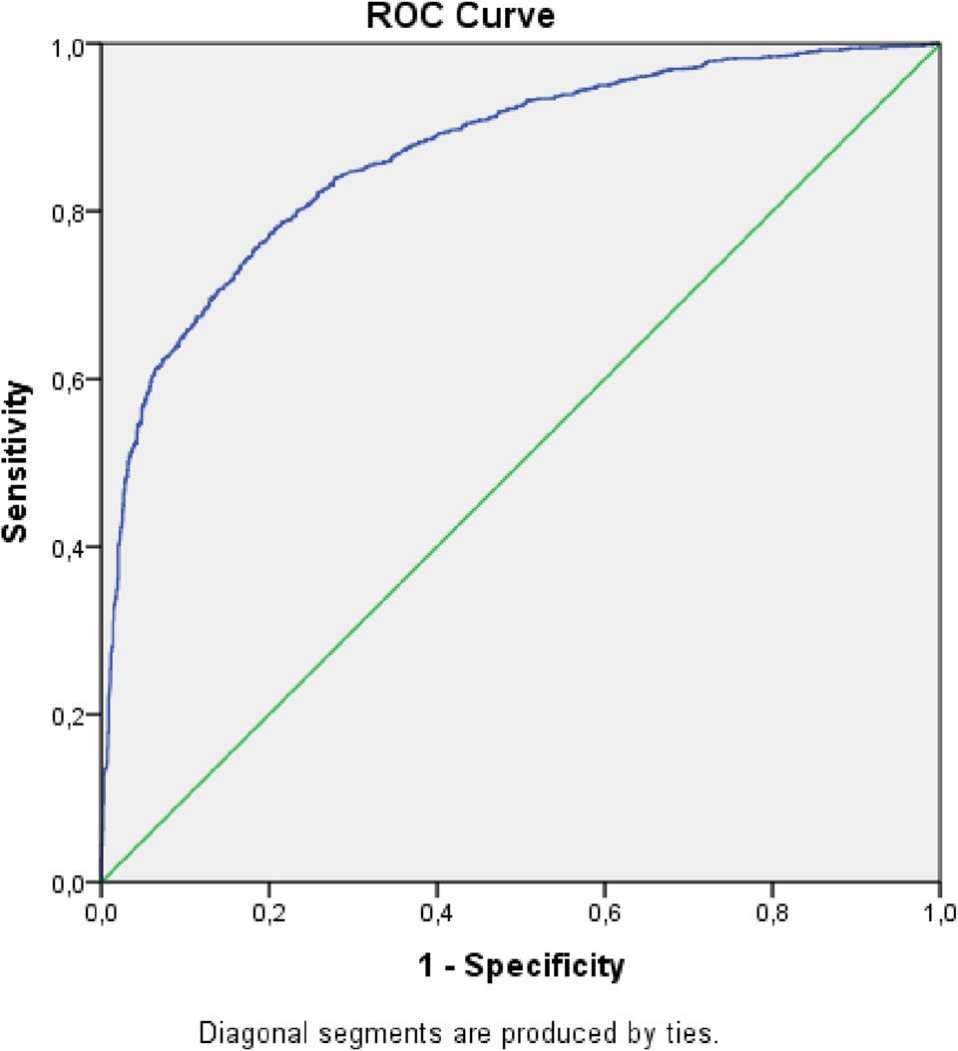
ROC curve of sensitivity and specificity of the multivariate model with CVD. Area Under the Curve, Test Result Variable(s): Predicted probability (area 0.867) (Std. error 0.06) (p-value < 0.01) (CI—0.855–0.879). Sensitivity (99.3%), Specificity (93.1%). Source: Ministry of Health, Sivep-Gripe/OpenDataSUS, 2020.

The ROC curve of the two multivariate models with and without CVD were satisfactory with area values above 80%: Model without CVD (Fig. 3)—Area Under the Curve, Test Result Variable(s): Predicted probability (area 0.833) (Std. error 0.04) (p-value < 0.01) (CI—0.873–0.892), Sensitivity (97.9%), Specificity (74.1%). Model with CVD (Fig. 4)—Area Under the Curve, Test Result Variable(s): Predicted probability (area 0.863) (Std. error 0.06) (p-value < 0.01) (CI—0.851–0.876), Sensitivity (99.3%), Specificity (93.1%). Thus the accuracy of the models with an area above 0.80 indicates that the model is adequate with regard to sensitivity and specificity of the regression for risk of death.

**Figure 5:**
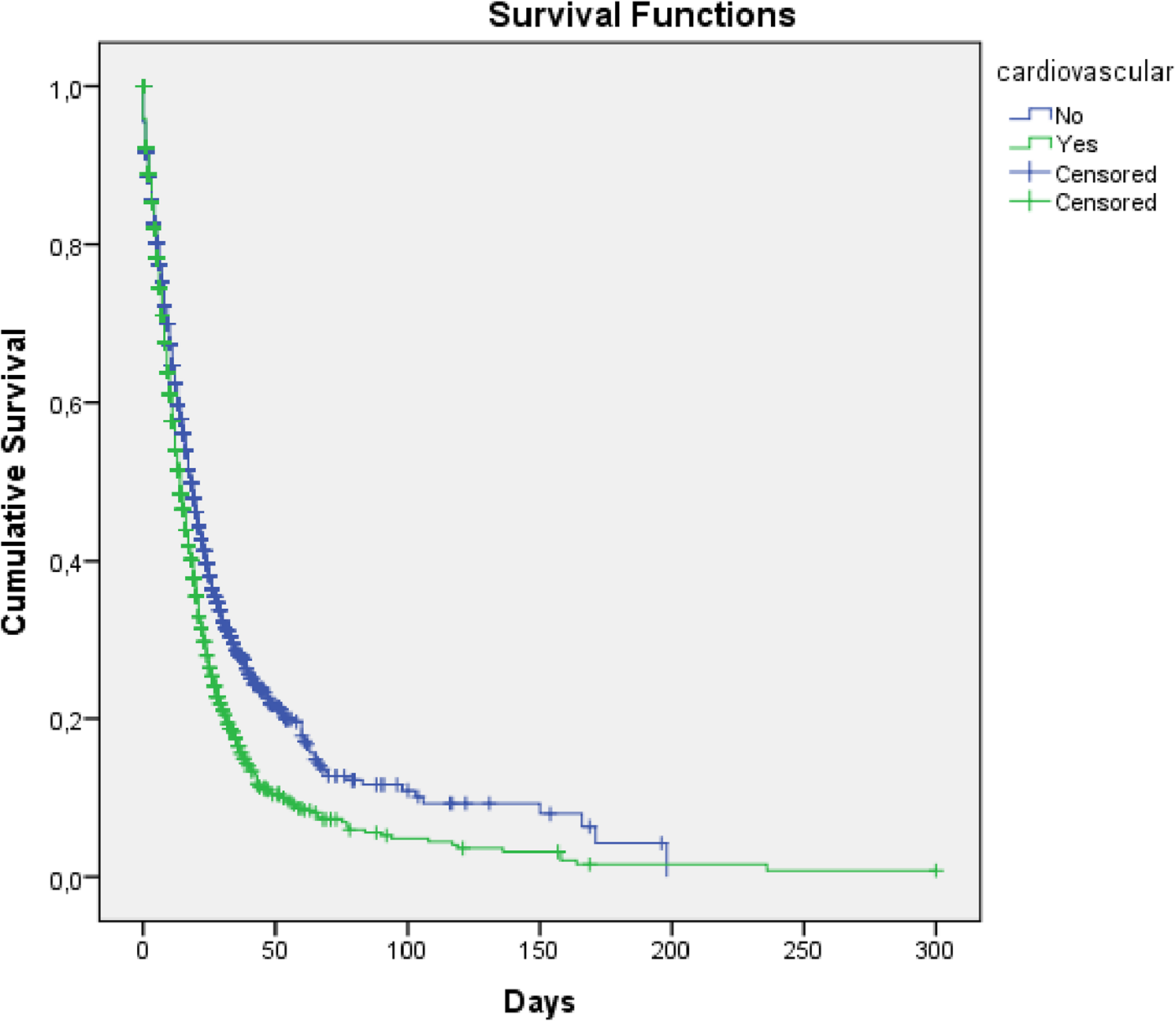
Survival analysis in patients with and without cardiovascular diseases until the outcome of death in cases of COVID-19 hospitalized in a region of the eastern Amazon in the first year of the pandemic, Pará state, Brazil, 2021. Long Rank (Mantel-Cox) (X^2^ 57.862—p < 0.0001), (2 log likelihood 58,300,403—p < 0.0001) Source: Ministry of Health, Sivep-Gripe/OpenDataSUS, 2020.

The survival analysis by the Kaplan–Meier test comparing cardiovascular disease patients with non-carriers, between the outcome survival (success) and death (failure), was significant according to the Long Rank test (mentel-Cox) (< 0.0001) showing statistical difference in the median of days, from the date of admission to the final outcome. For those without cardiovascular disease, the median number of days was (38—CI 34–46) in comparison to those with CVD (26—CI 23–30). This shows that patients with cardiovascular disease in severe COVID-19 in this study have a shorter survival in days comparison to non-carriers (Fig. 5).

## Discussion

As far as we know, this retrospective cohort is the first in Brazil and in the northern region of the country (eastern Amazon) to carry out a study of severe and hospitalized cases of COVID-19 with the dependent variable being a carrier of chronic cardiovascular diseases. We identified that the signs and symptoms of severity in association with lethality were significant in patients with CVD, as well as the cough and the outcomes ICU, invasive ventilation are predictors of lethality.

The literature has already well described that advanced age is a strong risk factor for death in COVID-19, as well as its association with comorbidities, which increase death in COVID-19^29–32^. Thus, age could be a confounding factor in our lethality analysis when we include all ages. We showed that lethality in CVD patients is higher than general mortality and in non-CVD patients, similar to the results of Zhang et al.^33^, however, in the analysis of lethality by age group, we showed that there was no difference in those older than 60 years, but in those younger than 59, there was a statistical difference in higher lethality in patients with CVD. Showing that CVD in those under 59 years of age is a predictor of lethality in this cohort.

In a Danish cohort of patients hospitalized with COVID-19, 22.1% had ≥ 1 CVD, 23.7% had severe infection within 30 days, and 12.6% died. The predicted risks of both outcomes at 75 years among men with single CVD comorbidities did not differ by clinically significant amounts compared to those without comorbidity risks for the composite endpoint of severe infection^34^, similar to our results when no difference was observed of lethality in the elderly. Another study identified a higher risk of death in elderly patients with CVD when compared to elderly non-carriers, but male, and associated with a lower number of leukocytes and higher levels of C-reactive protein^35^.

We identified in this study that the signs and symptoms that represent the severity of the disease and death were significant in patients with CVD, dyspnea, respiratory distress and an O_2_ saturation equal to or less than 95% as well as the presence of other comorbidities and risk factors such as diabetes and obesity. Regarding the outcome, invasive ventilation and ICU admission were the predictors of death, as well as the lower survival rate in patients with CVD. Similar to the results of a meta-analysis and meta-regression with 4448 hospitalized and critically ill COVID-19 patients identified that those with CVD were associated with an increased probability for a worse outcome (RR 2.23 [1.71. 0.91], p < 0.001; I 2: 60%), for mortality (RR 2.25 [1.53.3.29], p < 0.001; I 2: 33%) and for severity (RR 2.25 [1.51.3.36], p < 0.001; I 2: 76%). The Meta-regression showed that the association was not influenced by sex, age, hypertension, diabetes and respiratory comorbidities. Furthermore, the association between cerebrovascular disease and poor outcome was not affected by cardiovascular diseases and vice versa^36^.

A retrospective cohort of 288 adult patients compared CVD and non-CVID carriers in COVID-19 in relation to factors associated with ICU admission and showed that older age, dyspnea, increased troponin I, C-reactive protein and creatinine were associated in CVD patients to have higher chances of ICU admission reported in regression models^37^. An observational study in Korea with patients hospitalized with COVID-19 showed patients that went to the ICU (5.3% vs. 1.6%; P < 0.001) with invasive ventilation (4.3% vs. 1.7%; P < 0.001) and death (12.9% vs. 3.1%; P < 0.001) were significantly higher in patients with cardiovascular risk factors or preexisting CVDs^38^. Therefore, ICU admission, invasive ventilation and death are associated with CVD patients, results similar to our cohort.

It is worth noting that invasive ventilation represents the most severe phase of the disease, since it is an advanced treatment modality for severe hypoxemia^39–41^. In our Amazon region resources are limited regarding technologies for other non-invasive ventilation alternatives in the treatment of COVID-19 are used in other countries^42^. Another limitation of the study in relation to invasive ventilation is the different guidelines between countries for the indication of invasive ventilation and local technologies^43^. Thus we showed that the cases that underwent invasive ventilation died more, in both comparisons with CVD and without CVD, but the presence of CVD potentiates the chances of death in the severe case that required invasive ventilation.

Chronic kidney disease was the second predictor of death in patients with CVD presenting almost three times the chance of death. One study showed that chronic kidney disease predicts cardiovascular disease, and that the predictive relationship persists even after statistical adjustment for traditional cardiovascular risk factors, associated with several changes. For example, when glomerular filtration is below 60 ml per minute for individuals over 40 years of age there is an increase in cardiovascular risk^44^. Therefore, the relationship may be a hypothesis that chronic kidney disease is an important predictor of death in COVID-19 in patients with CVD in this cohort.

The cardiovascular morbidity rate in the participants of this cohort was 34.83% of the cases. Most of the patients were older, with a lower rate of cardiovascular morbidity than in a study in the United States in 2016 and 2017 in hospitalized patients with SARS diagnoses by other coronaviruses that presented a cardiovascular morbidity rate of 56%^45^. This is associated with the highest incidence of CVD in developed countries^46^. A meta-analysis of severe cases of SARS by MERS-CoV with 637 patients showed the incidence of CVD carriers around 30%^47^, an incidence approximate to that of our study.

A systematic review with meta-analysis with 48,317 hospitalized with COVID-19 that included observational studies from China, USA, Italy, UK and Iran showed an overall prevalence of CVD of 8.33% in the 44 studies (95% CI 6.32–10.91) and the prevalence increased with age, in which 4.51%, 9.26% and 15.02% of patients aged < 50 years, 50–60 years and ≥ 60 years, respectively, were found to have CVD. They highlighted that patients under 50 years of age had a higher risk of developing fatal outcomes (35.7% vs 10.9%, OR 5.66, 95% CI, 4.12–7.79) in comparison to those over 60 years of age. (37.1% vs 23.8%, OR 2.10, 95% CI 1.68–2.61)^48^. This is a much lower CVD morbidity rate than our cohort in this region from the Amazon, however, the results were similar in the evidence of greater lethality in patients with CVD under 60 years of age with COVID-19.

In Brazil a study before the pandemic by COVID-19, evaluated the factors associated with SARS in a southern state of the country, referring to cases notified in 2015 and 2017, with 3,340 notified of all etiologies, the prevalence of cardiovascular comorbidity in those hospitalized for SARS was (487/14.7%)^49^. Another study in Brazil analyzed the cases of SARS in hospitalized patients in a state in the southeastern region of the country, and identified a prevalence of (21.4%) of patients with cardiovascular diseases who were notified for SARS in 2019 before the pandemic. However, when the same study analyzed in 2020 the prevalence of cardiovascular diseases in those notified for SARS, the results were higher (48.8%), but it is worth noting that the analysis referred to all etiologies of SARS, COVID-19 had a prevalence of (54.2%) in this cumulative analysis^50^. A study in the same region of the Amazon analyzed the risk factors for severity of COVID-19, but included all cases, mild, moderate and severe, totaling 100,819 being the largest epidemiological study of COVID-19 conducted in the region in the period from March 1 to July 29, 2020. Regarding the risk of death from cardiovascular disease, the study showed in the logistic regression model (OR 4.090—CI 3.759–4.450) a four times greater chance of death in patients with CVD^25^.

Another meta-analysis with COVID-19 hospitalized patients also showed a lower rate of cardiovascular morbidity among patients 12.89%, with studies from China, USA, Italy, Korea, Singapore and Iran that gathered 77,317 cases of severe COVID-19^51^.

In this study, we show that the cardiovascular morbidity rate in severe COVID-19 is higher than in other published studies on COVID-19, and we also confirm the higher fatality rate in the < 1 to 59 year age groups in CVD patients compared to those without CVD.

Some limitations of this study should be noted as several cases were excluded due to lack of data in the forms, as well as the risk of bias in filling in the variable with or without CVD, which could have increased the CVD morbidity rate in this research. It is also noteworthy that the surveillance of SARS in Brazil has been strengthened since the influenza pandemic in 2009, and it is a protocol in the country to collect biological material for molecular surveillance of the etiologies in cases of SARS in hospitalized patients, as well as the surveillance of risk factors such as CVD that have been included in the notification and investigation form since 2009 when surveillance was boosted in the country. Another limitation is the weakness of local epidemiological surveillance, since due to the large numbers of hospitalized SARS cases, surveillance prioritized the investigation, closure of cases, and entry into the surveillance platform of cases that went on to die, leaving healing cases in the background, which may explain the higher lethality rate than other studies. But it is worth noting that the local risk factors for deaths in general in this region are already higher than in other regions of Brazil, in relation to infectious causes.

## Conclusions

In this cohort of severe cases of COVID-19 in the northern state of Pará, Brazil (eastern Amazon), we identified that the signs and symptoms of severity and lethality already described in the literature are associated with greater potency in patients with CVD, signifying that COVID-19 has a less favorable outcome in patients with CVD in this study.

We would also like to state that the cardiovascular morbidity rate among patients with severe COVID-19 in this cohort is higher than the rates reported in the literature in patients hospitalized for COVID-19.

The lethality in cases over 60 years of age was not significant, unlike younger patients aged < 1 to 59 years who had a higher rate of mortality, demonstrating that being a carrier of CVD in COVID-19 is an independent predictor of death. Additionally, the known predictors of death in COVID-19 are increased in relation to the probability that a patient has CVD, with the exception of cough, which has been shown to be a predictor of death directly associated with patients with CVD.

## Data Availability

The datasets generated during and/or analysed during the current study are available in the [OpenDataSUS] repository, [https://opendatasus.saude.gov.br/dataset/srag-2020].
